# The Oxidant Role of 4-Hydroxynonenal in Corneal Epithelium

**DOI:** 10.1038/srep10630

**Published:** 2015-05-29

**Authors:** Longlong Chen, Rongrong Zong, Jing Zhou, Lianping Ge, Tong Zhou, Jian-xing Ma, Zuguo Liu, Yueping Zhou

**Affiliations:** 1 Eye Institute and affiliated Xiamen Eye Center of Xiamen University, Fujian Provincial Key Laboratory of Ophthalmology and Visual Science, Xiamen, Fujian, 361005, PR China; 2 Department of Physiology, University of Oklahoma Health Sciences Center, Oklahoma City, Oklahoma 73104, USA

## Abstract

4-Hydroxynonenal (4-HNE or HNE) is a main endogenous product of cellular lipid peroxidation in tissues and is reported to play pathogenic roles in eye diseases. Here we investigated the association between 4-HNE and oxidative stress in the corneal epithelium. 4-HNE suppressed the cell viability of human corneal epithelial cells (HCE) in a concentration dependent manner. 4-HNE significantly increased the level of 3-Nitrotyrosine (3-NT), a marker of oxidative stress, in HCE cells and corneal epithelium of rats by immunofluorescent staining and Western blot analysis. To its underlying mechanistic on ROS system, 4-HNE elevated the ROS generation enzyme NADPH oxidase 4 (NOX4) and induced the activation of NF-E2–related factor-2 (NRF2) and its downstream effectors: NAD(P)H dehydrogenase (quinone 1) (NQO1) and glutathione S-transferase P (GSTP). Furthermore, N-acetylcysteine (NAC), an antioxidant and ROS scavenger, antagonized the inhibitory and oxidant effects of 4-HNE on the corneal epithelial cells. In conclusion, 4-HNE plays an oxidant role in the corneal epithelium and this work provides a new strategy for the pathogenesis and treatment of corneal diseases.

Oxidative stress refers to an imbalance between oxidation and antioxidation in the cells, which is considered to be an important factor leading to aging and diseases. The main products of oxidative stress are reactive oxygen species (ROS), such as superoxide anion (O_2_^−^), hydrogen peroxide (H_2_O_2_), hydroxyl radical (·OH), and so on^1^. These free radicals and superoxides can attack the polyunsaturated fatty acids (PUFA) in the phospholipid membrane of cells, which causes lipid peroxidation and forms a great diversity of aldehydes^2^.

4-Hydroxynonenal (4-HNE or HNE) is a main product generated during lipid peroxidation. 4-HNE is an alpha beta unsaturated aldehyde (OαβUAs) formed in the lipid peroxidation process of ω-6 PUFA in cells such as linoleic acid, linolenic acid and arachidonic acid^3^. 4-HNE at higher concentration can react with some nucleophilic substances, such as sulfhydryl compounds, DNA, protein and phospholipid and so on, and induce dysfunction of cells, which results in cell damage and diseases. On the other hand, 4-HNE at lower concentrations is also a bioactive molecule regulating multiple cell signaling pathways and gene expressions, and is involved in cell proliferation, necrosis and apoptosis^4^. Furthermore, 4-HNE, as a main product of lipid peroxidation, has become an important marker of lipid peroxidation/oxidative stress, and it has been widely applied in experimental research to induce cell oxidative stress or apoptosis^5^,^6^,^7^.

As surface organs, the eyes are directly exposed to light and environment, which make eye tissues more susceptible to a variety of physical and chemical factors that may lead to oxidative stress^8^. Extensive research suggests that oxidative stress plays vital roles in the pathogenesis of many ocular diseases. Multiple clinical and experimental studies demonstrated that 4-Hydroxynonenal is associated with some ocular surface diseases, including granular corneal dystrophy, atopic keratoconjunctivitis, pterygium, and dry eye^9^,^10^,^11^,^12^, as well as cataract^13^, age related macular degeneration (AMD)^14^ and diabetic retinopathy^15^, while the role and the underlying mechanism of 4-HNE in the eyes remain largely unknown. The aim of this present study was to focus on and investigate the association between 4-HNE and oxidative stress in the corneal epithelium and explore the underlying mechanistic.

## Materials and Methods

### Materials

4-Hydroxynonenal (4-HNE or HNE) was purchased from Enzo Life Sciences (Farmingdale, NY, USA). The Cell Counting Kit-8 (CCK-8) was purchased from Dojindo (Kyushu, Japan). The antibodies of anti-3NT, anti-NOX4, anti-NRF2, and anti-NQO1 were purchased from Abcam (Cambridge, United Kingdom). AlexaFluor488-conjugated IgG was purchased from Invitrogen (Carlsbad, CA, USA). The HRP-conjugated IgG antibody was purchased from Cell Signaling Tech Inc (Danvers, MA, USA). The antibody specific for β-actin and N-Acetylcysteine (NAC) were purchased from Sigma-Aldrich (Saint Louis, MO, USA).

### Cell Culture

Human corneal epithelial (HCE) cells, simian virus 40 transformed, were obtained from RIKEN Biosource Center, Tokyo, Japan, and were passaged in Dulbecco’s Modified Eagle Medium: Nutrient Mixture F-12 (DMEM-F12) media (Invitrogen, Carlsbad, CA, USA) supplemented with 6% heat-inactivated fetal bovine serum (FBS), bovine insulin (7 μg/mL), recombinant human epidermal growth factor (7 ng/mL), and 1% penicillin and streptomycin.

For cell viability assay, the HCE cells were plated at a density of 8000 cells per well in 96-well culture plates. When the HCE cells were cultured to 70% confluency, the media were removed and changed to DMEM-F12 basic media (serum free) containing different concentrations of 4-HNE (0.01, 0.1, 1, 5, 10, 20, and 100 μM) or the same amount of vehicle (n-hexane) and then cultured for 24 hours. For all other experiments, the HCE cells were plated at a density of 30 × 10^4^ cells per well in 6-well or 5 × 10^4^ cells per well in 24-well culture plates, cultured in specific concentrations of 4-HNE for 24 hours and followed by Western blot, quantitative real-time PCR assay, immunestaining following the procedures described below respectively.

In the N-Acetylcysteine (NAC) antagonization experiment, HCE cells were pretreated with different concentrations of NAC (0, 0.5, 1, and 2 mM) for 1 hour, then exposed to vehicle alone (n-hexane) or 4-HNE (20 μM) after transferred to fresh DMEM-F12 basic media (serum free) for another 24 hours for cell viability assay. For the immunostaining and Western blot assays, HCE cells were pretreated with NAC (1 mM) for 1 hour and then cultured with vehicle alone (n-hexane) or 4-HNE (20 μM) for 24 hours, respectively.

### *In Vivo* Experimental Procedures

Wistar rats (male, 180-220 g) were purchased from Shanghai Shilaike Laboratory Animal Co., Ltd., Shanghai, China. The animal experiments were carefully performed in accordance with the ARVO Statement for the Use of Animals in Ophthalmic and Vision Research and the animal experimental protocol was approved by the Animal Ethics Committee of Xiamen University School of Medicine (approval ID: XMUMC: 2013-04-20). The animals were kept in an air conditioned facility, with food and water ad libitum.

The rats were randomly divided into three groups (n = 6): (1) control group without any treatment (saline containing same amount of vehicle) ; (2) 4-HNE at lower concentration of 100 μM (HNE-L) group and (3) 4-HNE at higher concentration of 250 μM (HNE-H) group. The volume of topical administration was 10 μL. The administration was given at both eyes of rat twice daily at 0 and 12 hours, respectively. After 24 hours, the rats were sacrificed, followed by removal and dissection of the eyeballs or cornea. The dissected eyeballs were stored in a −80 °C freezer for the use of histologic and immunofluorescent staining. The method of dissection of corneal tissue for Western blot and quantitative real-time PCR was previously reported^16^. The whole corneal tissue was carefully dissected immediately under a surgical microscope by an experienced person.

### Cell Viability Assay

Cell viability was measured using Cell Counting Kit-8 (CCK-8) assay and conducted following the protocol of the manufacturer. After HCE cells were cultured for 24 hours in the conditional media, then the conditional media were replaced by CCK-8 constituted in culture media, followed by incubation for 4 hours at 37 °C in the dark. The solution was detected directly after incubation. The absorbance was measured spectrophotometrically at 450 nm with a Bio Tek ELX800 microplate reader (Bio Tek Instruments, Winooski, VT, USA).

### Immunofluorescent Staining

For the immunofluorescent staining with anti-3NT, anti-NOX4 and anti-NRF2 antibodies, the HCE cells were fixed in 4% paraformaldehyde for 1 hour and then kept in 70% ethanol for use, the corneal sections were fixed in cold acetone for 10 minutes. The HCE cells or the corneal sections were incubated with anti-3-NT antibody (1:150), anti-NOX4 antibody (1:150) and anti-NRF2 antibody (1:150) at 4 °C overnight, respectively. After three washes with PBS, The HCE cells or the corneal sections were further incubated in AlexaFluor488-conjugated IgG (1:300) for 1 hour. After thorough washes with PBS, the HCE cells or the corneal sections were mounted with VECTASHIELD Mounting Medium with 4`,6-diamidino-2-phenylindole (DAPI) (Vector, Berlingame, CA, USA), and photographed with a Leica DM2500 microscope (Leica microsystems, Wetzlar, Germany).

### Western Blot Analysis

The treated HCE cells or dissected corneal tissues were lysed and total cellular protein concentrations were measured by BCA Protein Assay Kit (Thermo, Waltham, MA, USA). Equal amounts of protein were resolved by electrophoresis through10% Tris-glycine SDS polyacrylamide gel and electrotransferred onto a PVDF membrane. The membrane was blocked with 1% (wt/vol) bovine serum albumin (BSA) in Tris-buffered saline with 0.1% Tween-20 (TBST) for 1 hour and subsequently incubated overnight at 4 °C with anti-3NT antibody (1:300), anti-NOX4 antibody (1:500) and anti-NRF2 antibody (1:500) at 4 °C overnight. After three washes with TBST, the membrane was incubated for 1 hour with a 1:10000 dilutions of an HRP-conjugated IgG antibody in TBST containing 1% BSA. After three washes with TBST, the bands were detected using a Moecular Imager ChemiDoc XRS System (Bio-Rad, Hercules, CA, USA). As needed, the membrane was stripped in stripping buffer (CWBIO, Beijing, China) and reblotted with an antibody specific for β-actin for loading control. The band intensities were semiquantified by densitometry using Quantity-One software.

### RNA Extraction and Quantitative Real-Time PCR assay

Total RNA was extracted from the dissected corneal tissues by using TRIzol reagent (Invitrogen, Carlsbad, CA, USA). Reverse transcription was performed with Oligo18T primers and reverse transcription reagents according to the manufacturer’s protocol (TaKaRa, Shiga, Japan). Quantitative real-time PCR was performed with mRNA special primers. The following primers were used for the PCR: for NRF2, 5′-AAACCAGTGGATCTGCCAAC-3′ (forward) and 5′-GACCGGGAATATCAGGAACA-3′ (reverse); for NQO1, 5′-CCATGAACTTCAATCCCATCAT-3′ (forward) and 5′-ACAGACTCGGCAGGATACTGAA-3′ (reverse); for GSTP, 5′-GCTCTATGGGAAGGACCAG-3′ (forward) and 5′-CTCAAAAGGCTTCAGTTGC-3′ (reverse). PCR reactions were performed on a BIO-RAD CFX-96 Real Time system (Bio-Rad, Hercules, CA, USA) with SYBR Premix Ex Taq (TaKaRa, Shiga, Japan) at 95 °C for 10 minutes, followed by 45 cycles of 95 °C for 10 seconds, 57 °C for 30 seconds, and 75 °C for 10 seconds, after which melt curve analysis was performed at once from 65 °C to 95 °C. All reactions were performed in triplicate and the average Ct (cycle threshold) values greater than 38 were treated as negative.

### Statistical Analysis

One-way analysis of variance test (GraphPad Software, Inc, La Jolla, CA, USA) was conducted to analyze the data from CCK-8 assay, Western blot, and quantitative real-time PCR, followed by a post hoc analysis Tukey test to compare the differences between the groups or a Student’s *t*-test. P value less than 0.05 was considered statistically significant.

## Results

### 4-Hydroxynonenal induces oxidative stress in the corneal epithelium

**Figure 1:**
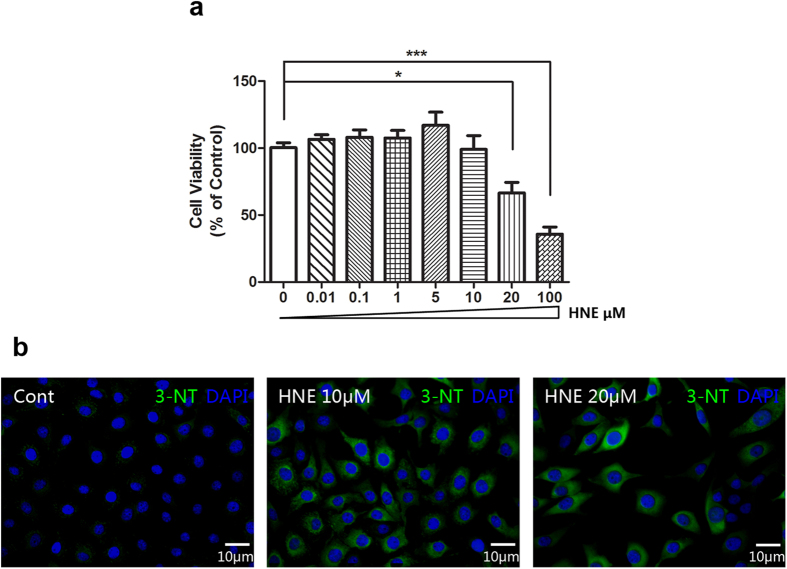
4-Hydroxynonenal induced oxidative stress in the cultured human corneal epithelial cells (HCE). **a:** Effects of 4-Hydroxynonenal (4-HNE or HNE) on the cell viability of HCE. 4-Hydroxynonenal at concentrations of 0, 0.01, 0.1, 1, 5, 10, 20, and 100 μM was treated for 24 hours. Data were presented as mean ± SEM; n = 4; *: p < 0.05, ***: p < 0.001. **b:** Representative images of immunofluorescent staining with anti-3-NT antibody after treatment of 4-Hydroxynonenal (10 and 20 μM) for 24 hours. Green color: 3-NT; Blue color: nuclear DAPI staining. Scale bar: 10 μm. (HNE=4-Hydroxynonenal).

We first applied different concentrations of 4-Hydroxynonenal (4-HNE or HNE) (0.01, 0.1, 1, 5, 10, 20, and 100 μM) in the cultured human corneal epithelial cells (HCE) to evaluate the inhibitory effects of 4-HNE on the cell viability. It showed that 4-HNE significantly suppressed the cell viability of HCE cells in a concentration dependent manner as concentration increased. (Fig. 1a). Meanwhile, the immunofluorescent staining images revealed that 4-HNE at 10 and 20 μM increased the expression of 3-Nitrotyrosine (3-NT), which is a marker of oxidative stress and is associated with corneal diseases, such as keratoconus, Fuchs’ dystrophy, and so on.^17^,^18^,^19^,^20^ (Fig. 1b), indicating that 4-HNE has oxidant activity on the corneal epithelial cells.

**Figure 2:**
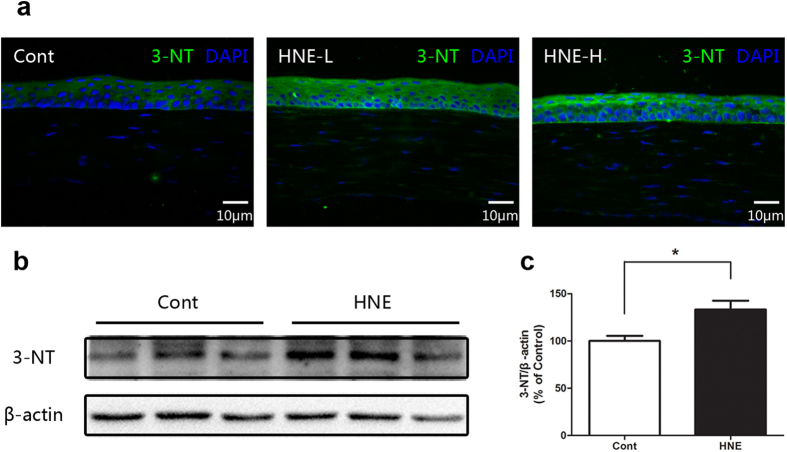
4-Hydroxynonenal induced oxidative stress in the corneal epithelium of rats. **a:** Representative images of immunofluorescent staining with anti-3-NT antibody after topical treatment of 4-Hydroxynonenal (HNE-L: 100 μM and HNE-H: 250 μM) for 24 hours. Green color: 3-NT; Blue color: nuclear DAPI staining. Scale bar: 10 μm. **b:** Representative images of Western blot with anti-3-NT antibody after topical treatment of 4-Hydroxynonenal (100 μM) for 24 hours. **C:** Statistical analysis of Western blot results. Data were presented as mean ± SEM; n = 3; *: p < 0.05. (HNE=4-Hydroxynonenal).

To confirm the oxidant effect of 4-HNE on corneal epithelium, we next performed topical administration of 4-HNE (100 and 250 μM) in rat eyes. It was demonstrated by immunofluorescent staining and Western blot that the level of 3-NT of corneal epithelium was significantly enhanced after treatment of 4-HNE (Fig. 2a-c), while the pathological and histological examination demonstrated that topical treatment of 4-HNE did not result in significant injury or damage to the corneal epithelium (data not shown).

### 4-Hydroxynonenal upregulates ROS generation enzyme: NOX4

To illustrate the mechanism of oxidant activity of 4-HNE, we first investigated the effects of 4-HNE on the ROS system, by focusing on NADPH oxidase 4 (NOX4), which is a key enzyme of ROS generation^21^,^22^,^23^,^24^.

**Figure 3:**
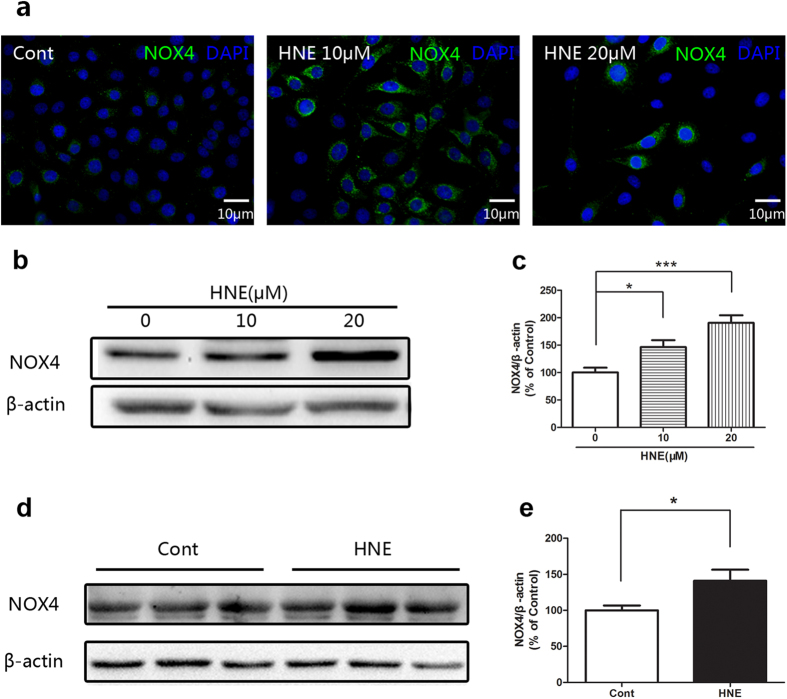
4-Hydroxynonenal upregulated the ROS generation enzyme NOX 4 in the cultured human corneal epithelial cells (HCE) and the corneal epithelium of rats. **a**: Representative images of immunofluorescent staining of HCE with anti-NOX4 antibody after treatment of 4-Hydroxynonenal (10 and 20 μM) for 24 hours. Green color: NOX4; Blue color: nuclear DAPI staining. Scale bar: 10 μm. **b**: Representative images of Western blot of HCE with anti-NOX4 antibody after treatment of 4-Hydroxynonenal (10 and 20 μM) for 24 hours. **c**: Statistical analysis of Western blot results of HCE. Data were presented as mean ± SEM; n = 7; *: p < 0.05 and ***: p < 0.001. **d**: Representative images of Western blot of corneal epithelium of rats with anti-NOX4 antibody after topical treatment of 4-Hydroxynonenal (100 μM) for 24 hours. **e**: Statistical analysis of Western blot results of rats. Data were presented as mean ± SEM; n = 3; *: p < 0.05. (HNE=4-Hydroxynonenal).

It was shown by immunofluorescent staining and Western blot that NOX4 was significantly upregulated in the cultured corneal epithelial cells after application of 4-HNE (Fig. 3a-c). Furthermore, the upregulation of NOX4 induced by 4-HNE was also supported by the *in vivo* experimental evidence, the Western blot data revealed that 4-HNE significantly increased the level of NOX4 (Figs. 3d, 3e). These results suggested that 4-HNE plays oxidant role in the corneal epithelium through upregulating the ROS generation.

### 4-Hydroxynonenal induces the activation of NRF2

NF-E2–related factor-2 (NRF2) is considered a major component of ROS signaling pathway and can be activated by inducers in the event of oxidative stress^25^,^26^,^27^,^28^. NRF2 results in the induction of its downstream effectors including NAD(P)H dehydrogenase (quinone 1) (NQO1) and glutathione S-transferase P (GSTP)^29^,^30^. Next, we determined if 4-HNE induces the responses of NRF2.

**Figure 4:**
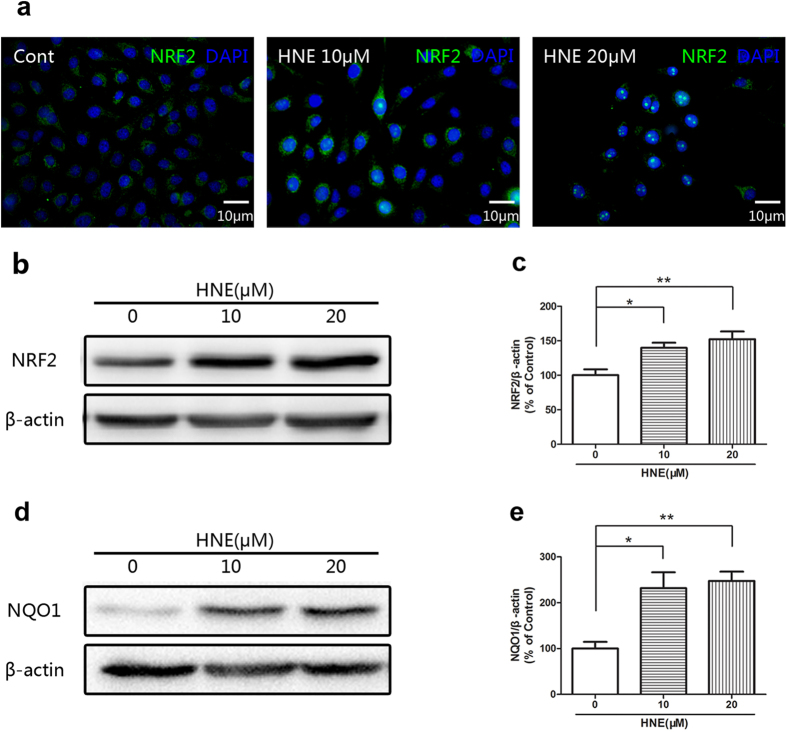
4-Hydroxynonenal induced the activation of NRF2 in the cultured human corneal epithelial cells (HCE). **a:** Representative images of immunofluorescent staining with anti-NRF2 antibody after treatment of 4-Hydroxynonenal (10 and 20 μM) for 24 hours. Green color: NRF2; Blue color: nuclear DAPI staining. Scale bar: 10 μm. **b:** Representative images of Western blot with anti-NRF2 antibody after treatment of 4-Hydroxynonenal (10 and 20 μM) for 24 hours. **c:** Statistical analysis of Western blot results of NRF2. Data were presented as mean ±  SEM; n = 10; *: p < 0.05, **: p < 0.01. **d:** Representative images of Western blot with anti-NQO1 antibody after treatment of 4-Hydroxynonenal (10 and 20 μM) for 24 hours. **e:** Statistical analysis of Western blot results of NQO1. Data were presented as mean ± SEM; n = 4; *: p < 0.05, **: p < 0.01. (HNE=4-Hydroxynonenal).

**Figure 5:**
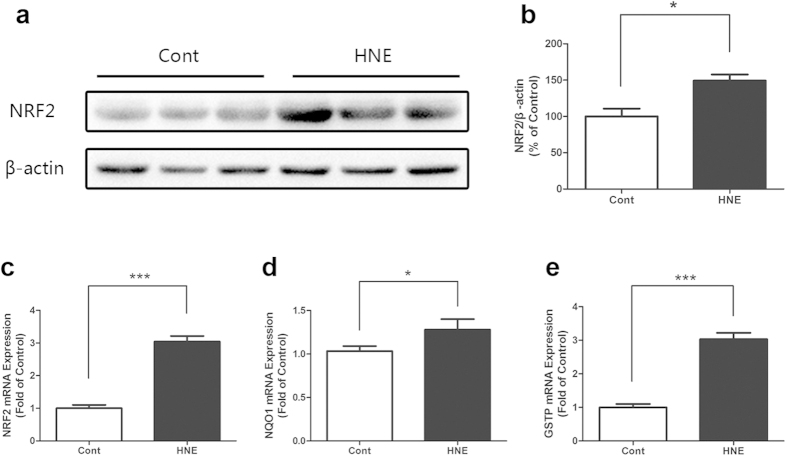
4-Hydroxynonenal induced the activation of NRF2 in the corneal epithelium of rats. **a:** Representative images of Western blot with anti-NRF2 antibody after topical administration of 4-Hydroxynonenal (100 μM) for 24 hours. **b:** Statistical analysis of Western blot results of NRF2. Data were presented as mean ± SEM; n = 3; *: p < 0.05. **c, d, e:** Statistical analysis of quantitative realtime PCR results of gene expressions of NRF2, NQO1 and GSTP after topical treatment of 4-Hydroxynonenal (250 μM) for 24 hours, respectively. Data were presented as mean ± SEM; n = 3; *: p < 0.05, ***: p < 0.001. (HNE=4-Hydroxynonenal).

It was demonstrated by immunofluorescent staining that 4-HNE enhanced the expression and accumulation of NRF2 in the cytoplasm and 4-HNE also induced the nuclear translocation of NRF2 in the corneal epithelial cells (Fig. 4a). The Western blot results also showed that 4-HNE significantly increased the level of NRF2 in the cultured corneal epithelial cells (Figs. 4b, 4c). Further, the level of NQO1 was significantly increased in the HCE after treatment of 4-HNE (Figs. 4d, 4e). On the other hand, the activation of NRF2 was revealed in the *in vivo* experiments. It was shown by the quantitative real-time PCR and Western blot that the topical treatment of 4-HNE significantly increased the gene expression of NRF2 and protein level of NRF2 (Fig. 5a-c) in the corneal epithelium. In addition, topical administration of 4-HNE significantly increased the gene expression of NQO1 and GSTP, which are downstream effectors of NRF2 (Figs. 5d, 5e).

Taken together, these data indicated that 4-HNE induces oxidative stress in the corneal epithelium via activation of NRF2 and its downstream effectors.

### N-acetylcysteine antagonizes the effects of 4-Hydroxynonenal

Finally we applied N-acetylcysteine (NAC), which is an antioxidant and ROS scavenger^31^ to antagonize the oxidant activity of 4-HNE on the cultured corneal epithelial cells.

**Figure 6:**
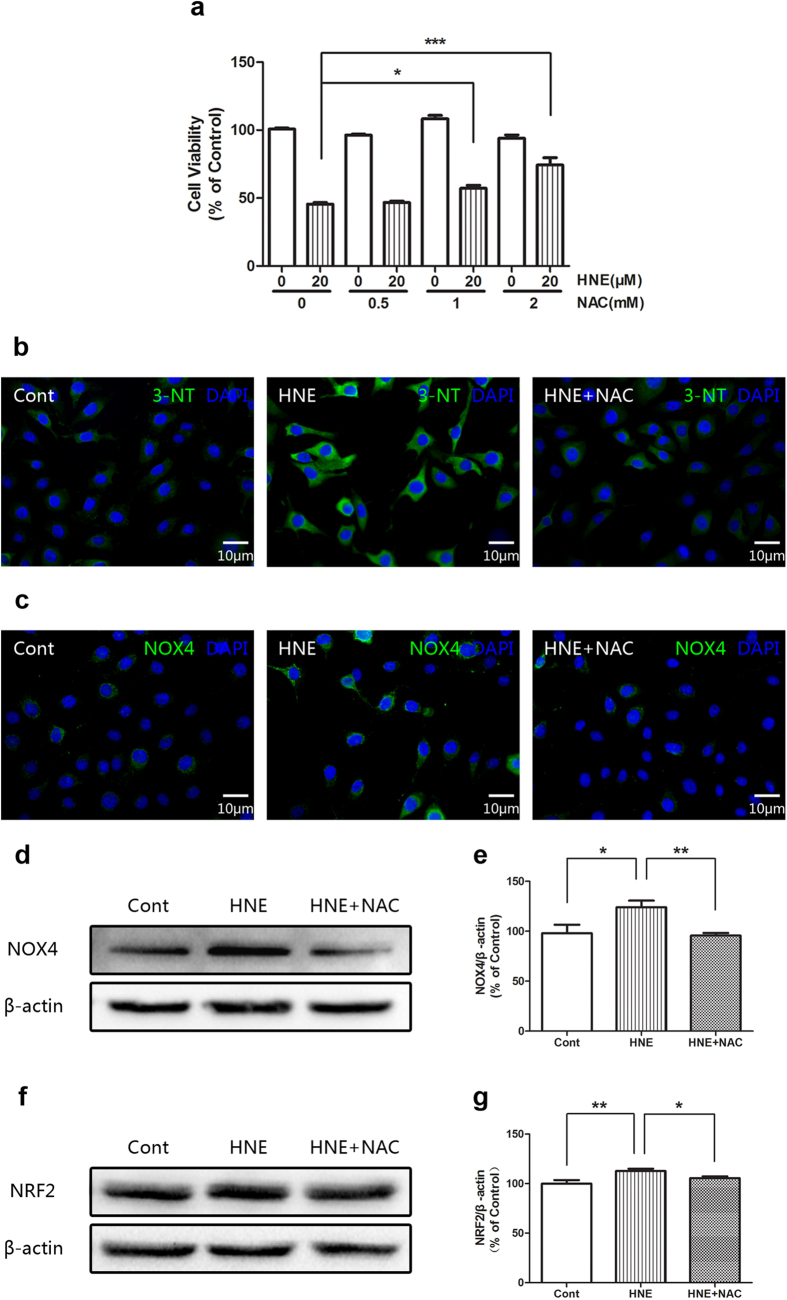
N-acetylcysteine (NAC) antagonized the effects of 4-Hydroxynonenal in the cultured human corneal epithelial cells (HCE). **a:** Effects of N-acetylcysteine (NAC) on the cell viability induced by 4-Hydroxynonenal. HCE cells were pretreated with NAC (0.5, 1 and 2 mM) for 1 hour and then were exposed to vehicle alone or HNE (20 μM) for another 24 hours, followed by CCK-8 assay. Data were presented as mean ± SEM; n = 5; *: p < 0.05, ***: p < 0.001. **b, c:** Comparisons of representative images of immunofluorescent staining with anti-3-NT (upper panel) and anti-NOX4 (lower panel) antibody between HNE (20 μM) only group and HNE (20 μM) plus NAC (1 mM) group after treatment for 24 hours, respectively. Green color: 3-NT or NOX4; Blue color: nuclear DAPI staining. Scale bar: 10 μm. **d:** Comparison of representative images of Western blot with anti-NOX4 antibody between HNE (20 μM) only group and HNE (20 μM) plus NAC (1 mM) group after treatment for 24 hour. **e:** Statistical analysis of Western blot results of NOX4. Data were presented as mean ± SEM; n = 3; *: p < 0.05, **: p < 0.01. **f:** Comparison of representative images of Western blot with anti-NRF2 antibody between HNE (20 μM) only group and HNE (20 μM) plus NAC (1 mM) group after treatment for 24 hour. **g:** Statistical analysis of Western blot results of NRF2. Data were presented as mean ± SEM; n = 5; *: p < 0.05, **: p < 0.01. (HNE=4-Hydroxynonenal).

It showed that N-acetylcysteine (NAC) reversed the inhibitory effects of 4-HNE on the cell viability of HCE cells in a concentration dependent manner (Fig. 6a). It was demonstrated by immunofluorescent staining that NAC reduced the immunostaining signals of 3-NT (Fig. 6b) of 4-HNE treated group, whereas the results of immunofluorescent staining and Western blot revealed that NAC blocked the increased level of NOX4 induced by 4-HNE (Fig. 6c-e). Furthermore, the Western blot data also showed that NAC antagonized the increased level of NRF2 induced by 4-HNE (Fig. 6f-g). These data suggested that the oxidant activity of 4-HNE is antagonized by NAC.

## Discussion

In the present investigation, we provided novel evidence that 4-Hydroxynonenal serves as an inducer to oxidative stress in the corneal epithelium via regulating the reactive oxygen species (ROS) system. Since 4-Hydroxynonenal is an endogenous product of lipid peroxidation in the cells, this study will help to better understand the potential roles of 4-Hydroxynonenal in the pathogenesis of ocular surface diseases and will also provide new clues of direction for the treatment of ocular surface diseases.

Oxidative stress plays vital roles in the pathogenesis of many diseases, including ocular surface diseases, such as keratoconus, Fuchs’ dystrophy, and so on^17^,^18^,^19^,^32^,^33^,^34^. Our novel evidence from this study supported the linkage between the product of lipid peroxidation and oxidative stress in the corneal epithelium. It will be helpful to find the key elements to induce oxidative stress in the corneal epithelium, which may provide new potential agents to diagnose and treat the oxidative stress related corneal diseases.

The reactive oxygen species (ROS) system is complicated^32^,^33^,^34^. The ROS system includes ROS generation, such as NOX4 and ROS signaling pathway: NRF2 pathway, which activates its downstream factors, such as NQO1, GSTP and so on^25^,^26^,^27^,^28^,^29^,^30^. Our experimental data suggested that 4-Hydroxynonenal plays oxidant role through targeting on the ROS generation and signaling pathway. Meanwhile, the oxidant activity can be suppressed by the classic antioxidant: N-acetylcysteine. However, it needs further illustration on the effects of 4-Hydroxynonenal on other factors of ROS system and the biochemical binding sites of the factors that 4-Hydroxynonenal targets on. It becomes important to find the key proteins or factors of oxidative stress, which will help to better understand the pathogenesis of oxidative stress related diseases and explore new therapeutic medications in the treatment of oxidative stress related diseases.

Many antioxidants have been used as dietary supplements and medication. Extensive research has been done on the mechanism of anti-oxidants and exploration of new effective antioxidants. It is important to have a good experimental model of oxidation and to find the key factors which induce oxidative stress in the eye research, including corneal research. In this study, we applied a classic antioxidant: N-acetylcysteine (NAC) and showed that NAC reversed the oxidant effects of 4-HNE, providing new additional experimental cellular and animal oxidant model for the research of oxidative stress and development of new anti-oxidants in the cornea.

4-Hydroxynonenal has been previously reported to be associated with ocular surface diseases^9^,^10^,^11^,^12^. The novel evidence of this present study demonstrated that 4-HNE contributes to oxidative stress in corneal epithelium. This study will provide additional experimental model to investigate the oxidative stress related corneal diseases and will also offer new directions of the pathogenesis, diagnosis and treatment of ocular surface diseases.

## Additional Information

**How to cite this article**: Chen, L. *et al.* The Oxidant Role of 4-Hydroxynonenal in Corneal Epithelium. *Sci. Rep.* **5**, 10630; doi: 10.1038/srep10630 (2015).
